# A novel colchicine-myricetin heterozygous molecule: design, synthesis, and effective evaluations on the pathological models of acute lung injury *in vitro* and *in vivo*

**DOI:** 10.3389/fphar.2023.1224906

**Published:** 2023-06-29

**Authors:** Zhiyue Li, Xueqin Yan, Jiangchun Wei, Liuyang Pu, Guanbao Zhu, Yongkai Cao, Zhanyan Liu, Yaqian Liu, Yan Li, Limin Li, Xinping Li, Zhengzhi Wu

**Affiliations:** ^1^ Shenzhen Institute of Translational Medicine, The First Affiliated Hospital of Shenzhen University, Shenzhen, China; ^2^ Wu Zhengzhi Academician Workstation, Ningbo College of Health Sciences, Ningbo, China; ^3^ Shenzhen Institute of Advanced Technology, Chinese Academy of Sciences, Shenzhen, China; ^4^ Graduate School, Guangxi University of Chinese Medicine, Nanning, China

**Keywords:** colchicine-myricetin hybrid, colchicine, bleomycin, inflammation, acute lung injury, neutrophil elastase

## Abstract

Acute lung injury (ALI) is an inflammatory condition and there are no effective treatments. A novel new compound----colchicine-myricetin hybrid (CMyrH) was herein designed and synthesized. To evaluate the activity of CMyrH in ALI, we used a bleomycin (BLM) induced BEAS-2B injury model *in vitro* and established a well-recognized rat model of BLM-induced lung injury *in vivo*. The results demonstrated that colchicine-myricetin hybrid protected BEAS-2B cells against BLM-induced cell injury in an increased dose manner, and reduced wet/dry weight ratio, histological scoring, and inflammation cytokines IL-1β, IL-6, IL-18, and TNF-α levels of lung tissue of the rats. Furthermore, we found colchicine-myricetin hybrid inhibited caspase-1, ASC, GSDMD, and NLRP-3 expression *in vivo*. Meanwhile, we used molecular docking to analyze the binding mode of colchicine-myricetin hybrid and human neutrophil elastase (HNE), it revealed that colchicine-myricetin hybrid showed strong binding affinity toward human neutrophil elastase when compared to its parent molecules. In conclusion, It is suggested that colchicine-myricetin hybrid antagonized acute lung injury by focusing on multi-targets via multi-mechanisms, and might be served as a potential therapeutic agent for acute lung injury.

## 1 Introduction

Acute lung injury (ALI) is a life-threatening clinical inflammatory condition characterized by lung vascular permeability, pulmonary edema, massive alveolar damage, and recruitment of numerous inflammatory cells to the lungs. Loss of control over the migration of inflammatory cell infiltrations toward the inflamed lung tissue shares in the pathology of ALI and its more severe form, acute respiratory distress syndrome (ARDS) (Zhan et al., 2022; Hafez et al., 2023). Despite improvements in general supportive treatment and ventilatory care strategies designed to limit lung injury, no specific pharmacological therapy has yet proven to be efficacious in the management of acute lung injury (ALI) or ARDS. The most common causes of ALI are pneumonia and sepsis, despite recent progress, there is still a lack of knowledge on revealing pathological mechanisms of this condition (Bandela et al., 2022). Multiple studies have been performed to better elucidate the underlying pathogenic mechanisms of ALI. Examples of mechanisms explored in preclinical models of ALI include NOD-like receptor family pyrin domain-containing 3 (NLRP3) inflammasome and neutrophil elastase (NE) signaling. NLRP3, a key component of the inflammasome, plays a critical role in the secretion of IL-1β and in pyroptosis, which is an inherently inflammatory caspase-1-dependent mechanism of cell death triggered by various pathological stimuli that have been critically implicated in the pathogenesis of acute lung injury (Peiró et al., 2017; Hsu et al., 2022). NE is an active protease released from neutrophils involved in tissue damage by inducing direct cytotoxicity and proinflammatory mediator release (Ishida et al., 2023). Numerous evidence suggests that NE acts as a key target in ALI, and inhibitors of this protease are able to inhibit ALI in patients with cardiopulmonary bypass as well as other animal models associated with ALI (Pan et al., 2023). Bleomycin (BLM), originally designed as an anti-cancer drug, has now been demonstrated to cause ALI in multiple *in vitro* and *in vivo* experiment paradigms (Albanawany et al., 2022; Li et al., 2023; Lv et al., 2023). As such, BLM is widely accepted as an ALI inducer for identifying lung-protective compounds (Su et al., 2019; Chen et al., 2022). Intratracheal bleomycin administration induces acute lung inflammation and epithelial cell injury, followed by epithelial cell repair and fibrotic reactions (Chung et al., 2019). Herein, we used the bleomycin model of ALI to assess the effectiveness of CMyrH in the pathology.

Herb medicine is a great source from which a lot of drugs have been developed. (−)-Colchicine 1) is an iso-quinoline alkaloid isolated from medicinal plants including *Colchicum autumnale L.* and *Gloriosa superba L.* (Misra et al., 2023). Originally, colchicine was characterized as a mitotic inhibitor and universally applied in the treatment of cancer. In 2009, colchicine was approved by US FDA to treat gout and other related disorders. More encouragingly, colchicine has been proposed as a beneficial drug in the treatment of coronavirus disease 2019 (COVID-19) due to its excellent anti-inflammatory actions (Kacar et al., 2020; Pascual-Figal et al., 2021; Kasiri et al., 2023; Perricone et al., 2023). Whereas colchicine turned out to be an effective anti-inflammatory and anti-cancer agent, colchicine is still in the preclinical stages of development due to its high toxicity and severe side effects, including severe diarrhea, neuropathy, rhabdomyolysis, and bone marrow suppression (Spasevska et al., 2017; Kacar et al., 2020). Myricetin 2) is a flavonol compound existing in fruits and berries and has been reported to exert numerous pharmacological activities including antiviral, antioxidation and anti-tumor (Chaves et al., 2022; Sedbare et al., 2022). Myricetin is also well-recognized for its anti-inflammatory properties that act by inhibiting the formation of the NLRP3 inflammasome, which plays a key role in the innate immune response and the pathogenesis of many inflammatory diseases (Skrajnowska et al., 2021). At present, myricetin is listed as a natural health product and dietary supplement in Europe and has been approved by the FDA for pharmaceuticals, foods, and health products in the United States (Liu et al., 2020).

**FIGURE 1:**
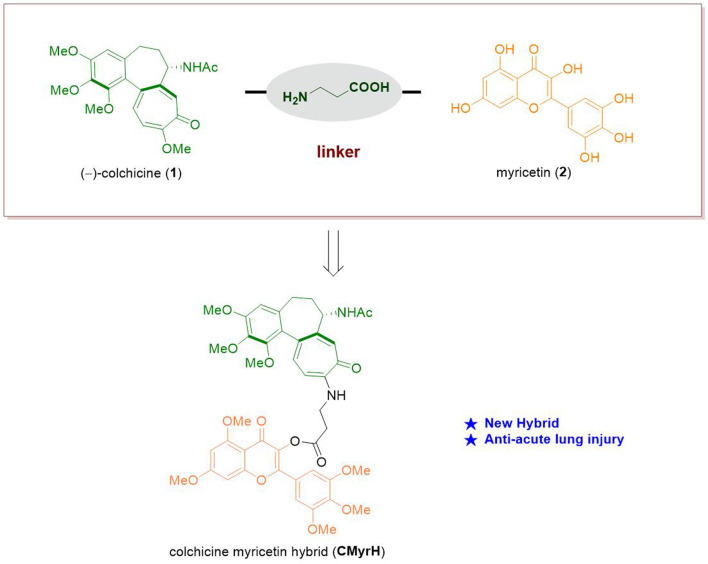
The design of a new hybrid of colchicine (1) and myricetin (2).

Since that, both colchicine and myricetin are able to counteract inflammatory response simultaneously, and myricetin possesses excellent safety, the hybridization of colchicine and myricetin may produce additive effects and reduce adverse and even toxic effects to treat complicated inflammatory diseases where ALI is involved. We have recently conducted a series of works exploring new drug candidates based on the concise synthesis and modification of (−)-colchicine 1) (Li et al., 2022; Pu et al., 2022). Herein we put forward our conception that the synthesis of a new hybrid of colchicine and myricetin to find a new bioactive molecule applied in anti-acute lung injury (Figure 1).

## 2 Materials and methods

### 2.1 Chemistry

General Information. Reaction mixtures at temperatures higher than ambient temperature were heated in an oil bath. All general chemicals were used directly without further purification. Petroleum ether (PE) refers to the fraction boiling in the 60°C–90°C range. Melting points were detected with an RY-I apparatus and uncorrected. NMR spectra of new compounds were recorded on a Bruker AV 400 spectrometer at 400 MHz (1H NMR), and 101 MHz (13C NMR). Chemical shifts were reported in ppm relative to internal TMS for 1H NMR data, and deuterated solvent for 13C NMR data, respectively. Data are recorded in the following format: chemical shift, multiplicity, coupling constant in hertz (Hz), and signal area integration in natural numbers. High-resolution mass spectra (HRMS) were obtained on Bruker Maxis impact instrument with ESI ion source and TOF mass analyzer.

### 2.2 Synthesis of CMyrH

To the solution of myricitrin (0.8 g, 1.72 mmol) in MeCN (40 mL), was added K_2_CO_3_ (1.8 g, 12.9 mmol) and dimethyl sulfate (1.2 mL, 12.9 mmol). The reaction mixture was refluxed for 24 h, then filtrated and concentrated in a vacuum. The obtained residue was dissolved in EtOH (40 mL), then was added conc. HCl (1.4 mL). The solution was stirred at 70°C for 12 h, then concentrated in a vacuum to remove EtOH. The residue was dissolved in DCM (40 mL) and washed with a saturated NaCl solution. The organic phase was dried over Na_2_SO_4_ and then concentrated *in vacuo* to yield crude product 4.

Compound 5 was synthesized according to the reported procedure (lit.). To the solution of compound 4 (620 mg, 1.6 mmol) and 5 (767 mg, 1.68 mmol) in DCM, was added EDCI (460 mg, 2.4 mmol) and DMAP (20 mg, 0.16 mmol). The solution was stirred at 25°C for 12 h. The contents were adsorbed on silica gel. The product was purified by column chromatography with EA to give the product CMyrH (595 mg, 45% yield) as a light yellow solid. MP 182°C–184°C. ^1^H NMR (400 MHz, CDCl_3_) δ 7.53–7.43 (m, 3H), 7.41 (d, *J* = 11.1 Hz, 1H), 7.05 (s, 2H), 6.63 (d, *J* = 11.3 Hz, 1H), 6.55 (d, *J* = 2.2 Hz, 1H), 6.51 (s, 1H), 6.40 (d, *J* = 2.2 Hz, 1H), 4.71–4.64 (m, 1H), 3.95 (s, 3H), 3.94 (s, 3H), 3.92 (s, 3H), 3.91 (s, 3H), 3.89 (s, 3H), 3.88 (s, 6H), 3.62 (s, 3H), 3.08 (t, *J* = 7.4 Hz, 2H), 2.46 (dd, *J* = 13.3, 6.5 Hz, 1H), 2.36 (td, *J* = 12.9, 6.7 Hz, 1H), 2.28–2.18 (m, 1H), 1.98 (s, 3H), 1.94–1.80 (m, 3H). ^13^C NMR (101 MHz, CDCl_3_) δ 175.47, 170.48, 170.07, 168.84, 164.74, 161.30, 159.34, 153.84, 153.40, 153.04, 151.45, 151.42, 151.24, 141.66, 140.70, 139.11, 134.68, 134.08, 130.99, 126.95, 124.81, 123.68, 108.75, 108.33, 107.32, 105.77, 104.98, 96.41, 92.88, 61.56, 61.45, 61.15, 60.56, 56.55, 56.48, 56.26, 56.04, 52.68, 38.51, 37.25, 33.31, 30.21, 23.01, 14.34. HRMS (*m/z*): calculated for C_44_H_46_N_2_NaO_14_ [M + Na]^+^) 849.2841, found 849.2859.

### 2.3 Drugs and chemicals

Colchicine (Q107209-15g) was purchased from Chemxyz Bio-technology Co., Ltd. (Shanghai, China), and myricetin (MED80097-25g) was from MedBio Pharmaceutical Technology Inc., (Shanghai, China). Bleomycin (B5507-15un) was from Sigma (St. Louis, MO, USA). Dulbecco’s modified Eagle’s medium (DMEM), fetal bovine serum (FBS), and other cell culture supplements were from Gibco (Grand Island, NY, USA).

### 2.4 Cell culture and treatment

Human bronchial epithelial cells (BEAS-2B cells, purchased from Procell, Wuhan, China) were incubated in DMEM with 10% FBS under the conditions of 5% CO2 at 37°C. BEAS-2B cells were passaged (dilution, 1:3) when attaining 90% confluency. For the establishment of models, BEAS-2B cells were disposed of diverse concentrations (0.01, 0.1, 0.3, 1, 3, 10, and 30 μg/mL) of BLM for 24 h. For the evaluation of protection of CmyrH, myricetin and colchicine, cells were pre-treated with CMyrH (0.01 μM, 0.1 μM, 0.3 μM, 1 μM, 3 μM, 10 μM), myricetin (0.01 μM, 0.1 μM, 1 μM, 3 μM, 10 μM, 30 μM, 100 μM) or colchicine (0.01 nM, 0.1 nM, 1 nM, 0.01 μM, 0.1 μM, 0.3 μM) for 2 h, and then exposed to BLM for 24 h.

### 2.5 Cell viability analysis

CCK-8 assay was applied to evaluate the cell viability of BEAS-2B cells. Briefly, BEAS-2B cells (2500 cells/well) were seeded into 96-well plates. The next day, cells were pre-treated with CMyrH, myricetin or colchicine for 2 h, and therewith undergo BLM for 24 h. At the end of treatment, CCK-8 solution (Fluorescence, Beijing, China) was directly pipetted into the culture medium (100 μL per well) and cells were further incubated at 37°C for 1 h. The absorbance was measured at 562 nm using a microplate reader.

### 2.6 Animals and care

Adult male-specific pathogen-free Sprague-Dawley rats (200 ± 20 g, 6–8 weeks) were purchased from the Guangdong VRLAT Co., Ltd. (Certificate: SYXK (Guangdong) 2022–0063). All rats were housed in standard cages in a climate-controlled room (24°C) under 12/12 h of light/dark cycles for 5 days. The experimental protocol was approved by the Animal Experimentation Ethics Committee of the Shenzhen Second People’s Hospital, protocol number 202300110. All the experimental procedures were carried out in accordance with the international guidelines for the care and use of laboratory animals.

### 2.7 Rat BLM-induced acute lung injury model

Acute lung injury was induced by airway delivery of bleomycin (BLM, 5 mg/kg) in rats, applying a previously described method (Albert et al., 2020; Zhou et al., 2020). Briefly, the trachea was exposed by making a small midline incision on the neck following anesthesia with isoflurane, and inoculated with 5 mg/kg bleomycin solution in 0.1 mL of PBS using a sterile syringe with a 28-gauge needle. Survival of rats were then checked three times daily for a period of 3 days.

### 2.8 Rat groups and drug treatment

Rats were weighed and randomly divided into six groups (9 rats per group). Rats from Col-treated groups were treated with different doses of colchicine (1.0 and 1.5 mg/kg, i.p.) and the CMyrH-treated groups with CMyrH (1.0 and 3.0 mg/kg, i.p.). Control and ALI-model rats were treated with saline only. Rats were euthanized with an overdose of 5% isofluorane inhalation 3 days after treatment, followed by cervical dislocation after lung tissues and blood samples harvesting.

### 2.9 Lung wet/dry weight ratio

At the endpoint, rats were sacrificed, the right upper lungs were removed, weighed (wet weight), and dried at 55°C for 72 h. The resulting dry lungs were weighed (dry weight), and the ratio between the two values was determined, without correction for blood content.

### 2.10 Lung pathology

The extracted lungs were histopathologically examined and assessed for inflammatory changes. For each group of rats, the left lobe of the lung of 4/8 rats was fixed in 4% of paraformaldehyde, and fixed sections of paraffin-embedded lungs were stained with hematoxylin and eosin (H&E). Lung lesions were scored at four levels (from 0 (normal), 1 (mild), 2 (moderate) to 3 (severe)) by the % of the affected tissue, as previously published (Glineur et al., 2014; Abdel-Daim et al., 2018; Qin et al., 2020).

### 2.11 Immunohistochemical staining

Immunohistochemical staining was performed on 4 mum paraffin sections prepared from the abovementioned resected specimens. Sections were incubated at 4°C overnight with a rabbit anti-caspase-1 polyclonal antibody (1:200, 22915-1-AP; Proteintech, Chicago, USA), a rabbit anti-ASC polyclonal antibody (1:100, GTX55818; Genetex, USA), a rabbit anti-NLRP-3 polyclonal antibody (1:200, bs10021R; Biosis, Beijing, China), a rabbit anti-GSDMD polyclonal antibody (1:200, 20770-1-AP; Proteintech, Chicago, USA) and a rabbit anti-NE polyclonal antibody (1:100, A13015; Abclonal, Wuhan, China) according to the manufacturer’s protocol. The IHC images were analyzed using a TEKsqray Digital Slide Scanner (SQS-40P, Shenzhen, China) and ImageJ software.

### 2.12 Inflammatory cytokines assay

Lung tissue lysates were prepared by homogenizing tissue in ice-cold lysis buffer containing 1 mM phenylmethane sulfonyl fluoride (PMSF), protease and phosphatase inhibitor cocktails (Servicebio, Wuhan, China), using a high-speed low-temperature tissue grinding machine (Servicebio, Wuhan, China), and then centrifuged at 13,000 rpm for 15 min at 4°C. The supernatant was collected and used to measure total protein concentration with the BCA assay (Solarbio, Beijing, China). The levels of inflammatory cytokines IL-1β, IL-6, IL-18 and TNF-α were measured by ELISA, using a Quantikine ELISA Kit (Spbio, Wuhan, China) and a microplate reader (Molecular devic, Guangzhou, China) at 450 nm, according to the manufacturer’s protocol, specific for each investigated cytokine.

### 2.13 Molecular docking and data processing

The crystal structure of neutrophil elastase (PDB ID: 6SMA) was obtained from the RSCB PDB database (http://www.rcsb.org/) and saved as a PDB format file. The structures of the proposed ligands were drawn through ChemDraw 15.0 and then converted to a 3D structure following energy minimization by Chem3D 16.0. The molecular docking was performed by Autodock 4.2, and binding energy was predicted to evaluate the affinity of the target proteins and active components. The best binding conformations were selected from 50 lowest energy docked structures based on a criterion combining the lowest binding energy and root-mean-square deviation values (RMSD <1) (Bahuguna et al., 2022), and were displayed by PyMOL. Here, if the binding energy of the ligand <0, the receptors bind spontaneously, and if the binding energy is ≤ −5.0 kcal/mol, they form a stable docking (Guo et al., 2022).

### 2.14 Statistical analysis

Statistical analyses were performed using GraphPad Prism Software (version 7.0 and 8.0). One-way ANOVA followed by posthoc tests, incorporating a Bonferroni correction for multiple comparisons, was employed, where *p* < 0.05 was considered significant. The cell viability and inflammatory cytokines were presented as mean ± SEM, and the lung W/D ratio was expressed as means ± SD. Cumulative survival rates were compared using the log-rank statistical analysis. Histological scores were expressed as median (interquartile range) and analyzed by the Kruskal–Wallis test followed by the Mann-Whitney U test.

## 3 Results

### 3.1 Chemical synthesis of CMyrH

**FIGURE 2:**
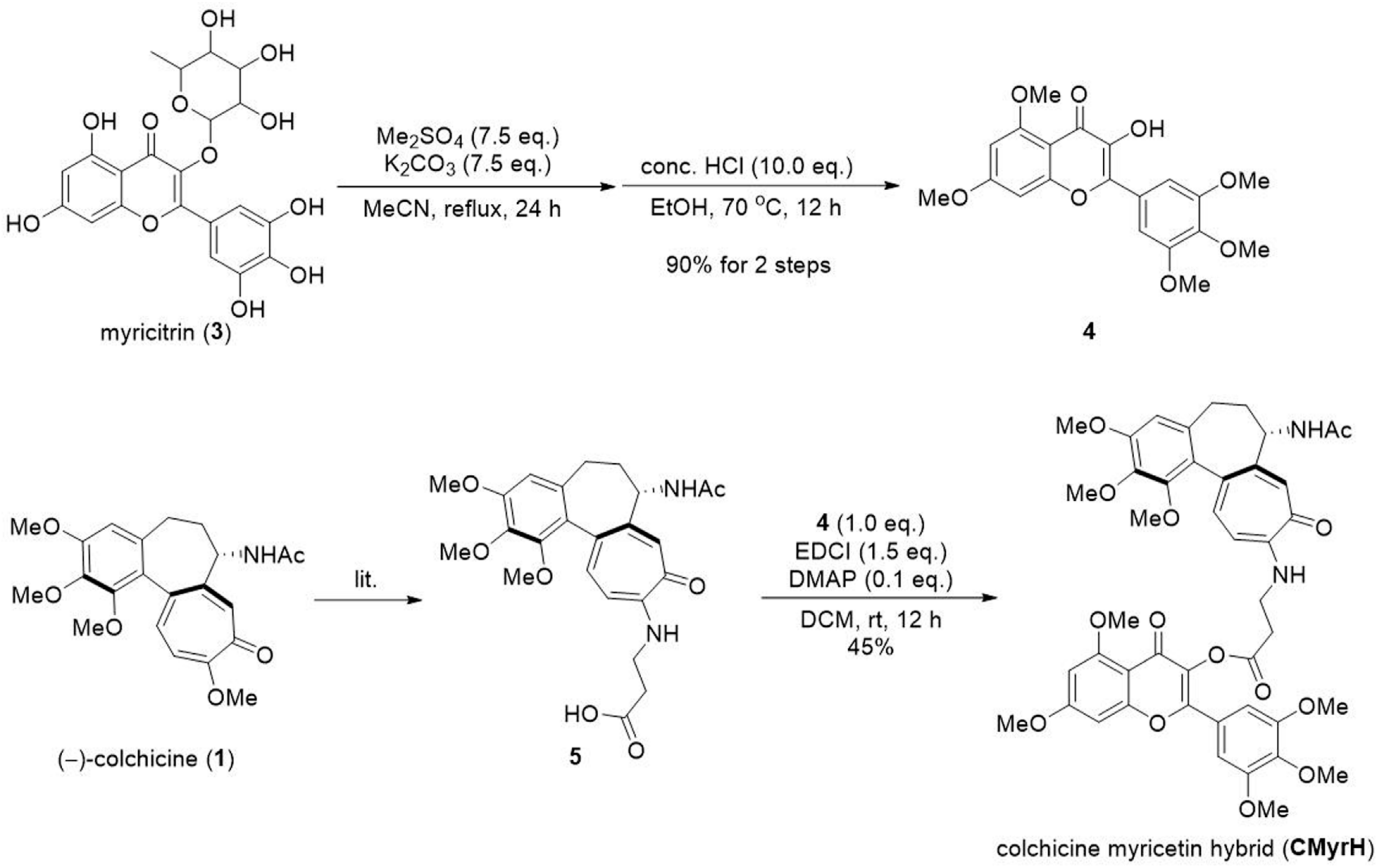
The synthesis route of CMyrH.

The synthetic method was shown in Figure 2. The myricitrin 3) was firstly methylated with excess dimethyl sulfate and Potassium carbonate. After being refluxed in MeCN for 24 h, the crude product was directly treated with HCl to afford the intermediate 4. Then compound 5 was synthesized from (−)-colchicine 1) in one step according to our reported procedure (Pu et al., 2022). Compounds 4 and 5 underwent an esterification reaction with EDCI and DMAP to provide the new hybrid CMyrH in moderate yield.

### 3.2 CMyrH alleviated BLM-induced BEAS-2B cell injury

**FIGURE 3:**
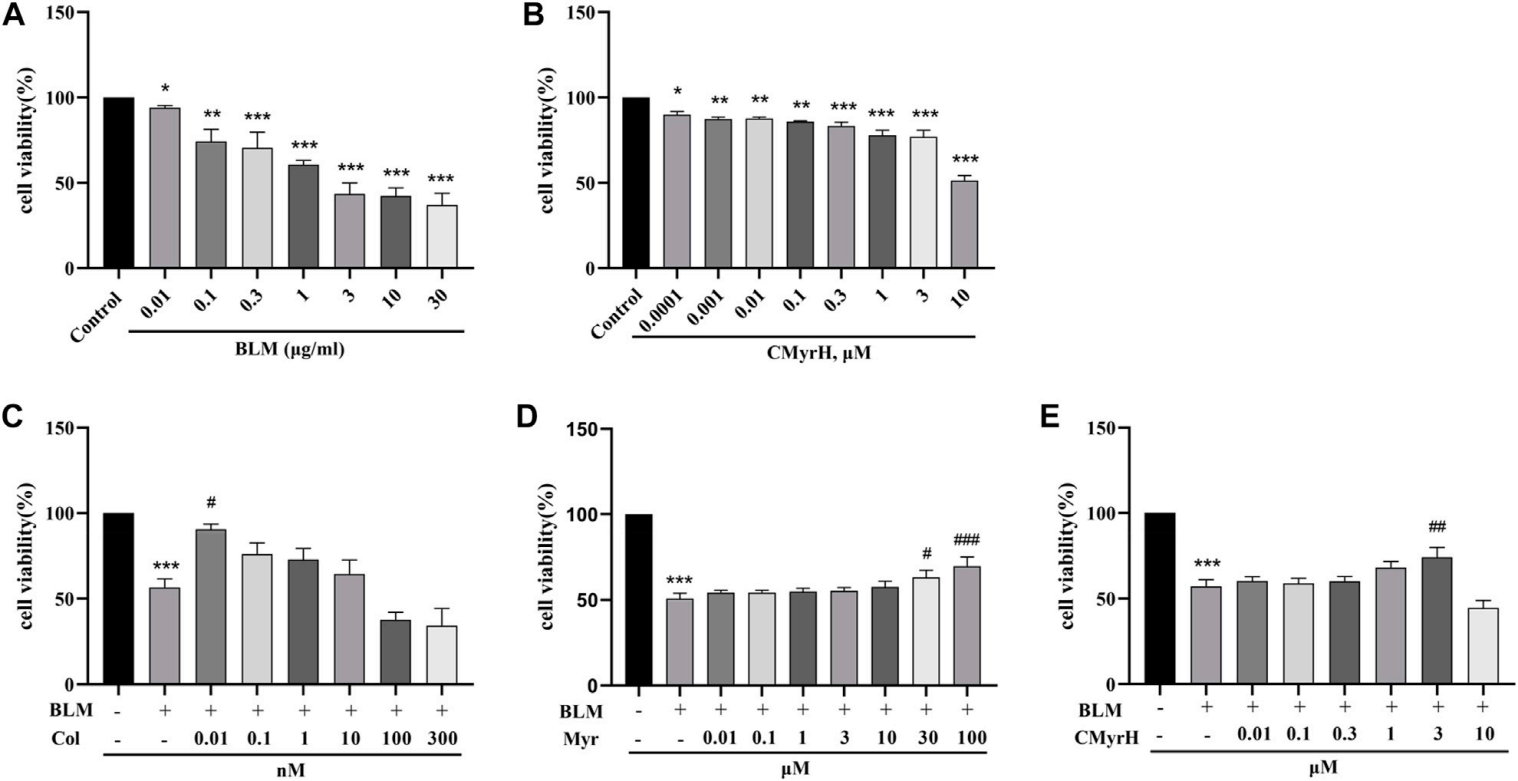
CMyrH protected against BLM-induced BEAS-2B injury. Effect of BLM **(A)** and CMyrH **(B)** treatment on BEAS-2B cell viability. Colchicine **(C)**, myricetin **(D)** and CMyrH **(E)** reversed the decrease in cell survival induced by BLM. All data are presented as mean ± SEM (n = 6 per group). ^*^ *p* < 0.05, ^**^ *p* < 0.01, ^***^ *p* < 0.001, *versus* control group; ^#^ *p* < 0.05, ^##^ *p* < 0.01, ^###^ *p* < 0.001, *versus* BLM group.

Since BLM at the dose of 10 μM resulted in approximately 50% cell death (Figure 3A), then 10 μM inducer was chosen for the subsequent experiments. To evaluate the protective effects of CMyrH, colchicine or myricetin against BLM-induced ALI, BEAS-2B cells were pretreated for 2 h with different concentrations of these compounds, then exposed to 10 μM BLM. It was found that CMyrH at 3 μM, colchicine at 0.01 nM, and myricetin at 30–100 μM protected BEAS-2B cells from BLM-induced injury (Figures 3C–E).

### 3.3 CMyrH decreased the toxicity of colchicine and improved the survival

**FIGURE 4:**
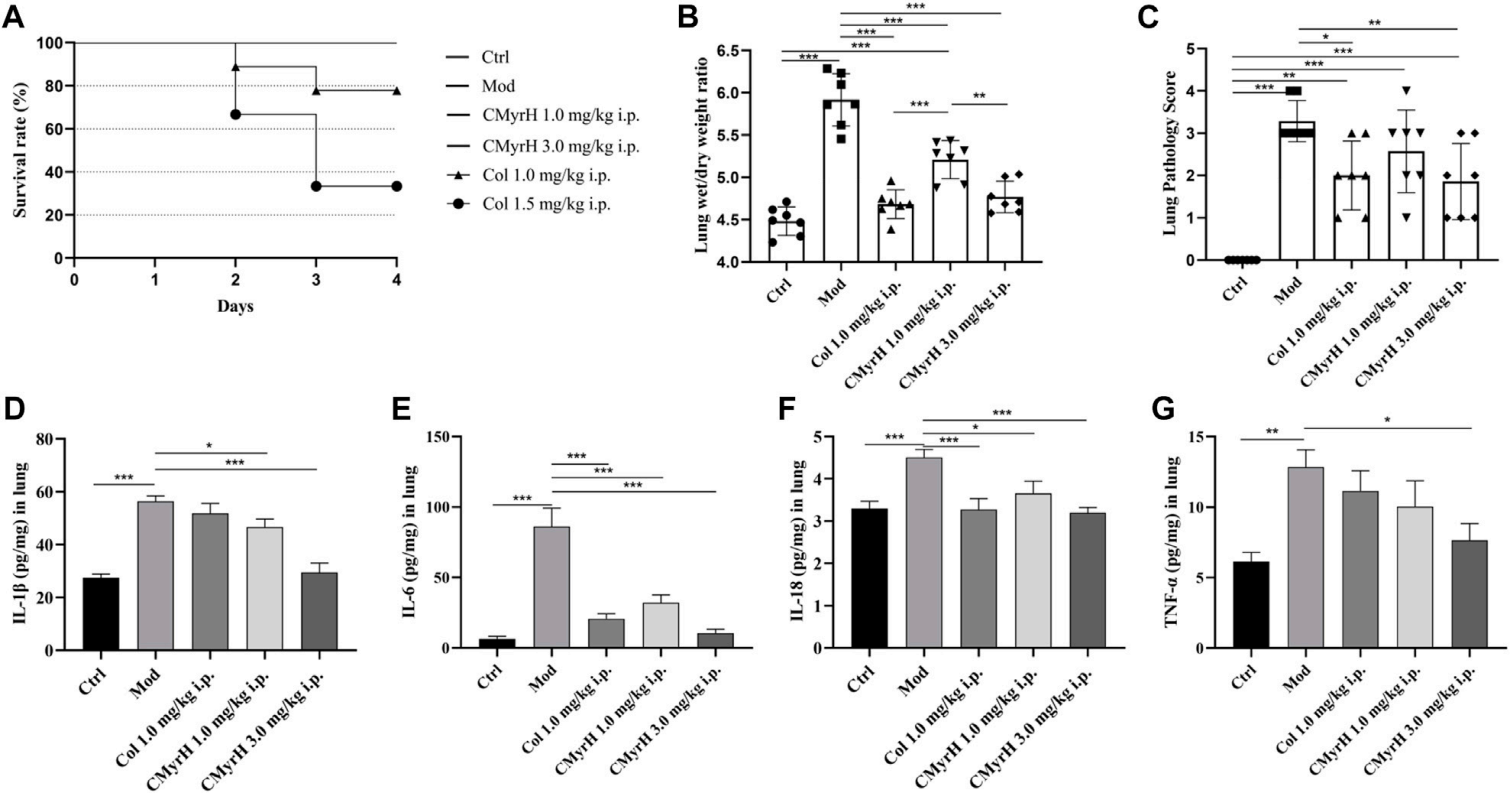
CMyrH improved survival, reduced pulmonary edema, restored pulmonary histology, repressed the production of proinflammatory cytokines, and was less toxic than colchicine. Panel 4**(A)**, the survival curve showed a significant difference in survival rate between BLM-challenged rats treated with colchicine or CMyrH. Panel 4 **(B)**, CMyrH reduced wet to dry lung weight ratio of lung tissues of rats at 72 h. Lung water content was determined in the right upper lobe (n = 7 per group) and measured via determination of lung wet-to dry-weight ratios, without correction for blood content. Panel 4 **(C)**, CMyrH downregulated histological scoring of lung tissues from BLM-induced rats following treatment. Lung lesions were scored from 0 to 3 based on a histopathologic scheme (n = 7). Panels 4 **(D–G)**, CMyrH alleviated the levels of IL-1β **(D)**, IL-6 **(E)**, IL-18 **(F)** and TNF-α **(G)** in the lung tissue of rats.

After an intratracheal BLM challenge, the survival rates of the model and CMyrH (1.0 mg/kg and 3.0 mg/kg, i.p.) groups remained at 100% within 72 h. However, we found that 6 out of 9 rats at colchicine doses of 1.5 mg/kg (vs. CMyrH 3.0 mg/kg i.p. group, log-rank test, *p* = 0.004) and 2 out of 9 rats at colchicine doses of 1.0 mg/kg (vs. CMyrH 1.0 mg/kg i.p. group, log-rank test, *p* = 0.145) were dead during 3 days of drug treatment (Figure 4A).

### 3.4 CMyrH decreased acute pulmonary edema in the ALI model

The lung wet/dry weight ratio (W/D) was elucidated to estimate pulmonary edema. Lung edema formation was significantly enhanced in the model group, as shown by an increased lung W/D (*p* < 0.001). All CMyrH and colchicine treatments led to a reduced lung W/D as compared to the model (*p* < 0.001), but no differences in the lung W/D were detected between COL- (1.0 mg/kg) and CMyrH- (3.0 mg/kg) treatment groups (Figure 4B).

### 3.5 CMyrH improved BLM-induced pathological alterations in lung tissue

**FIGURE 5:**
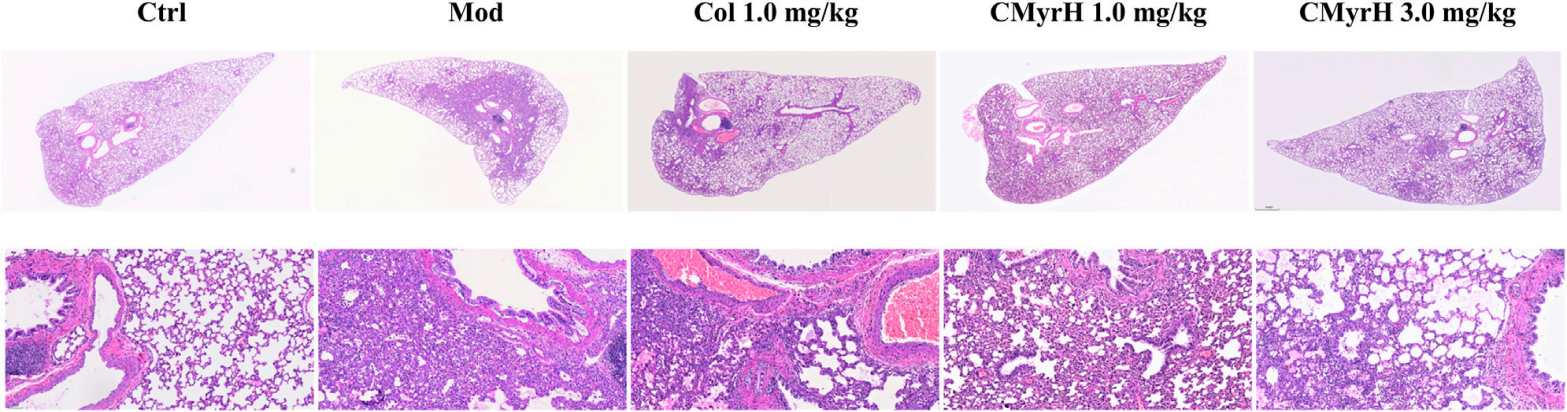
Microscopic pathological changes in the lung from treated rats. Lung sections (4 mum) were H&E stained with representative images shown from each group above (magnification ×40 and 200×).

Histologically, the lungs of BLM-induced rats showed moderate-to-severe pneumonia. CMyrH- (1.0 mg/kg) treated rats showed less severe (moderate) histopathological changes in the lungs. CMyrH- (3.0 mg/kg) and COL- (1.0 mg/kg) showed multifocal bronchiolitis and bronchitis to mild to moderate bronchointerstitial pneumonia (Figure 5). As shown in Figure 4C, median scoring of the severity of lung tissue damage at the end of the study demonstrated substantially reduced pathological scores in rats treated with 3.0 mg/kg CMyrH (*p* < 0.01) and 1.0 mg/kg colchicine (*p* < 0.05). The CMyrH- (1.0 mg/kg) treated rats also had reduced pathological median scores in lung tissues compared to the ALI-model rats, but this difference was not statistically significant (*p* < 0.05).

### 3.6 CMyrH attenuated BLM-induced lung inflammation via suppression of inflammatory cytokine expression

As shown in Figures 4D–G, the lung cytokine productions of IL-1β, IL-6, IL-18 and TNF–α in ALI-model rats were significantly increased postoperative administration of BLM. However, Treatments with both drugs (CMyrH and colchicine) mitigated the cytokine overproductions of IL-1β, IL-6, IL-18 and TNF-α, as compared to the ALI-model rats. The higher dose of CMyrH more effectively alleviated the inflammatory cytokines levels than the low dose.

### 3.7 CMyrH suppressed pyroptosis via inhibition of NLRP3, ASC, caspase-1, GSDMD expression

According to our research, the ALI-model group, when compared with the control group, showed elevated expression of NLRP3, ASC, caspase-1 and GSDMD, with an increase in the levels of IL-1β and IL-18. However, the groups which received CMyrH (1.0 and 3.0 mg/kg) exhibited a weak expression of caspase-1, ASC, GSDMD and NLRP3 accompanied by a reduction in IL-1β and IL-18 secretion in a dose-dependent manner than ALI-model rats. The present IHC analysis results suggested the ability of CMyrH to suppress pyroptosis caused by BLM in the rats’ lung tissues.

### 3.8 CMyrH inhibited neutrophil elastase activity by blocking the enzyme’s active site

The docking results provided the first evaluation as to which CMyrH exhibited a stronger binding affinity (higher docking score value) to NE, namely, CMyrH-b, which exhibited a higher affinity to NE than CMyrH-a, -c, and -d. Furthermore, silico studies showed that CMyrH inhibited NE protein by forming both a covalent bond between α-/β-unsaturated carbonyl groups in the colchicine fragment of CMyrH-b and Ser195, a hydrogen bond between the amino groups outside the 7-membered ring in colchicine fragment of CMyrH-b and Ser214, and a hydrogen bond between the ketone carbonyl groups in myricetin fragment of CMyrH-b and Gly216. Results showed that CMyrH has the highest binding energy values, −13.54 kcal/mol for NE protein (Ki value was 118.49 pM), followed by colchicine and myricetin with docking scores of −5.86 kcal/mol and −1.89 kcal/mol (Ki values were 51.03 μM and 40.93 mM), respectively.

## 4 Discussion

### 4.1 Efficient synthesis of a novel hybrid CMyrH

As for some adverse reactions, we altered the chemical structure of Colchicine to enhance its clinical application. Colchicine-CD44-Targeted Peptide conjugate decreased the cytotoxic effect of colchicine while maintaining anti-inflammatory against gout (Zoghebi et al., 2019). However, there were few studies about the hybridization of Colchicine with bioactive natural molecules to exert its efficient biological effects with low toxicity in ALI. In this current study, we designed a new kind of C-10-modified Colchicinoid which was a heterozygous molecule of Colchicine and Myricetin with a simple amino acid as the linker (Figure 2).

### 4.2 CMyrH caused lower toxicity and decreased BLM-induced BEAS-2B cells injury than colchicine

Subsequently, we used human lung epithelial BEAS-2B cells to estimate the effects of CMyrH and its parent molecules on BLM-induced cell injury. As shown in Figure 3, CMyrH antagonized significantly BLM-induced BEAS-2B cells injury, in a dose-dependent manner, especially in the 3 μM group; myricetin revised BLM-induced injury in increased dose-manner at 30 μM; colchicine reversed BLM-induced injury at 0.01 nM, but the safety range between the upper and lower limits was extremely narrow and even overlap, which creates difficulties when setting therapeutic margins. Thus, these results suggested that a novel hybrid CMyrH exhibited a better and safer anti-inflammatory effect on BLM-induced BEAS-2B cell injury compared with colchicine.

### 4.3 CMyrH ameliorated BLM-mediated inflammation in a ALI rat model

In the end, the CMyrH’s ability to improve ALI was further verified by using a rat BLM-induced acute lung injury model. As shown in Figures 4B–C and Figures 5H, E staining of lung tissue sections revealed less lung injury in rats administered CMyrH (at 1.0 mg/kg and 3.0 mg/kg) for 3 days post-challenge, with significantly lower lung injury score and W/D, indicating mild lung inflammation and pulmonary edema. CMyrH 1.0 mg/kg i.p. was relatively safe in rats up to 72 h, in addition, an acute toxicity study of CMyrH showed no signs of toxicity or cause mortality in rats even at doses = 3.0 mg/kg (Figure 4A). For reference, colchicine at 1.0 mg/kg reduced histopathological end scores and W/D in their lung tissues compared to the model rats (Figures 4B,C), however, colchicine administration for 72 h showed severe toxic effects with a 22.2%, 66.7% mortality rate at the doses of 1.0 mg/kg and 1.5 mg/kg i.p. (Figure 4A). Furthermore, multiple cytokines in lung tissue dramatically declined after treatment with the diverse concentration of CMyrH (1.0 mg/kg and 3.0 mg/kg), importantly, a high dose of CMyrH remarkably reversed proinflammatory cytokines including IL-1β, IL-6, IL-18 and TNF-α more than that of a low dose (Figures 4D–G). These findings indicated that threefold doses of CMyrH had already achieved anti-inflammation efficacy comparable or even superior to colchicine, without severe toxicity or treatment-related deaths.

### 4.4 CMyrH suppressed NLRP3 inflammasome-mediated pyroptosis caused by BLM in the rats’ lung tissues

Pyroptosis is a form of cell death triggered by the innate immune system that has been implicated in the pathogenesis of acute lung injury (Hsu et al., 2022; Liang et al., 2022). Activated NLRP3 recruits ASC and caspase-1 to form the NLRP3 inflammasome, resulting in the cleavage of gasdermin D (GSDMD) and membrane pores formation (pyroptosis) accompanied by the proinflammatory cytokines IL-1β and IL-18 release (Faria et al., 2021; Tummers and Green, 2022). In the current study, the immunohistochemical expression of caspase-1, ASC, GSDMD and NLRP3 was investigated in lung sections to reflect on the anti-pyroptosis potential of CMyrH. Our findings showed a decrease in caspase-1, ASC, GSDMD and NLRP3 immunoreactivity in lung sections accompanied by a reduction in cytokine production of IL-1β and IL-18 affiliated to both CMyrH-treated (1.0 mg/kg and 3.0 mg/kg) groups. These evidences suggested that CMyrH might protect rats against ALI caused by BLM by alleviating lung inflammation through the inhibition of pyroptosis.

### 4.5 CMyrH antagonized ALI by binding to the active site of the neutrophil elastase enzyme

**FIGURE 6:**
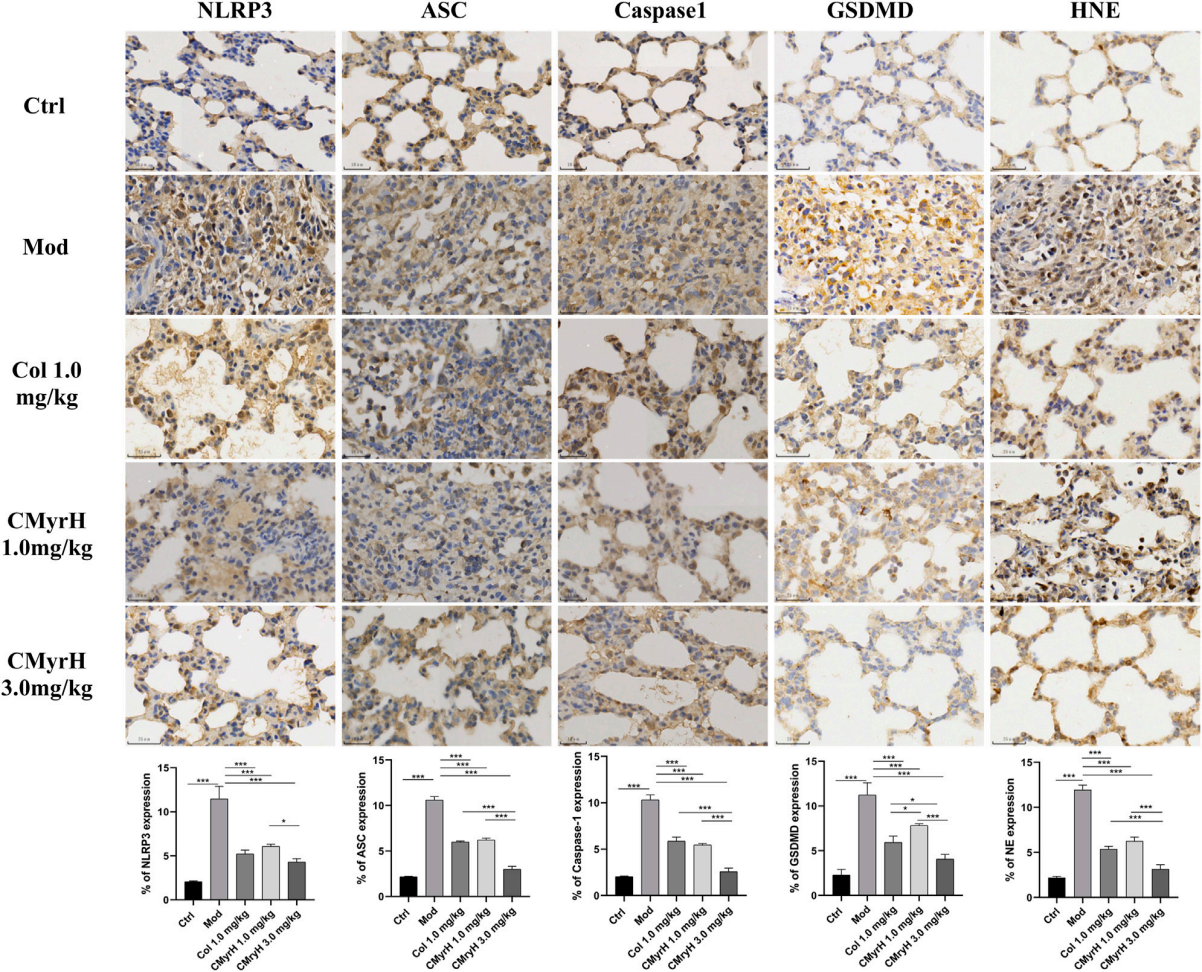
Effects of CMyrH on NLRP3, ASC, Caspase-1, GSDMD and HNE immunoreactivities in lung sections. The five upper lines, immunohistochemical detection of NLRP3, ASC, Caspase-1, GSDMD and HNE expression in lung tissues, x400. The bottom line, corresponding NLRP3, ASC, Caspase-1, GSDMD and HNE labeling index values. Values were presented as mean ± SD of 4 rats per group. ^*^ *p* < 0.05, ^**^ *p* < 0.01, ^***^ *p* < 0.001.

**FIGURE 7:**
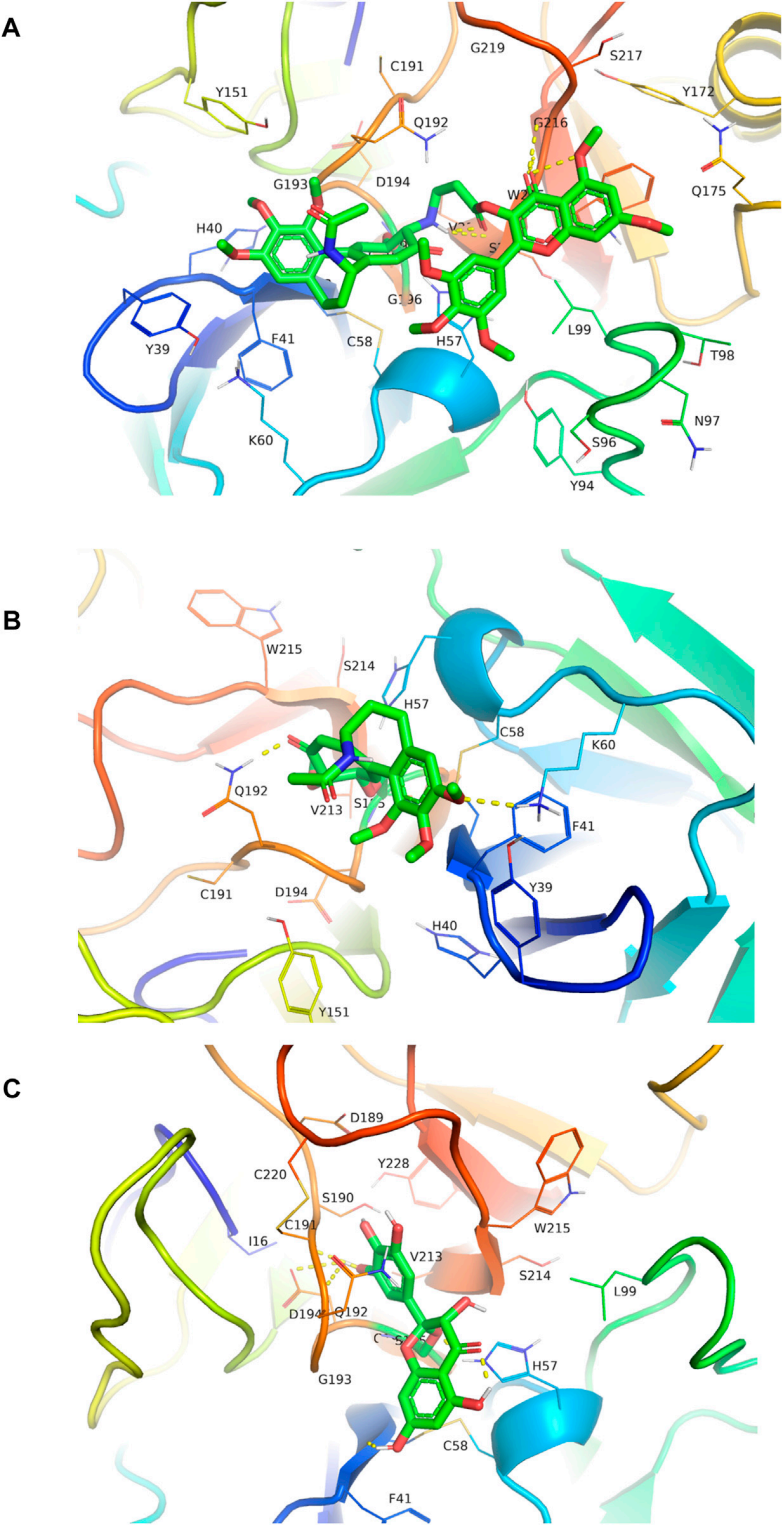
Molecular docked CMyrH panel **(A)**, colchicine panel **(B)**, and myricetin panel **(C)** into neutrophil elastase crystal structure (6SMA) using AutoDock 4.2. CMyrH bounded to amino acid residue Ser195 via a covalent bond and to Ser214 and Gly216 via two hydrogen bonds in the active site of NE protein. Among the three docking complexes, CMyrH has the highest binding energy values of −13.54 kcal/mol for NE protein (Ki value was 118.49 pM) as compared to others.

Human neutrophil elastase (HNE) is a potent serine protease secreted by activated polymorphonuclear neutrophils (PMNs), whose uncontrolled production can result in inflammatory-derived disease conditions. (González-Fernández et al., 2018.). Besides its crucial role in powerful host defense, HNE is well known as one of the most destructive enzymes in the human body (Thierry, 2020). It is reported that excessive HNE activity is involved in the pathogenesis of ALI, thereby administering anti-NE might prevent lung tissue damage caused by inflammation (Conrad and Eltzschig, 2020). Analyzing the resultant *in silico* interactions of the CMyrH-b molecule with the residues of host HNE protein showed that CMyrH-b molecule significantly hit most of the active site residues of the HNE protein with strong interactions, such as covalent bond and hydrogen bonding. The docking score with NE indicated that CMyrH was the highest affinity binding with the lowest energy value −13.54 kcal/mol (Ki value was 118.49 pM) as compared to other docking complexes. Immunohistochemical studies confirmed this result, with lung tissues from CMyrH-treated rats analyzed showing a decrease in immunoreactivity for antibodies specific to HNE (Figure 6 Figure 7). It is suggested that CMyrH-b had a strong inhibitory effect on neutrophil elastase, which might also be an important mechanism for CMyrH-b to antagonize ALI.

## 5 Conclusion

In conclusion, we herein designed and synthesized a novel hybrid CMyrH by splicing colchicine and myricetin. CMyrH exerted strong anti-inflammatory abilities to alleviate bleomycin-induced BEAS-2B cell injury, and to protect the lung from inflammatory injury and pulmonary edema in a rat model of ALI, even conferring a survival benefit. The present findings insinuated a superior potential of CMyrH over colchicine in inhibiting NLRP3 inflammasome-mediated pyroptosis. And the key to CMyrH’s pharmacological properties also included its stable binding to the active site in the NE enzyme. These findings obtained *in vitro* and *in vivo* models of bleomycin-induced ALI were confirming the promising therapeutic potential for ALI of novel hybrid CMyrH supporting their future employment as a novel inflammation strategy.

## Data Availability

The original contributions presented in the study are included in the article/Supplementary Material, further inquiries can be directed to the corresponding author.
