# Supplementary material for: A novel colchicine-myricetin heterozygous molecule: design, synthesis, and effective evaluations on the pathological models of acute lung injury in vitro and in vivo
Source: Front Pharmacol. 2023 Jun 29;14:1224906. doi: 10.3389/fphar.2023.1224906 (PMC10340118; doi:10.3389/fphar.2023.1224906)
Supplement: Supplementary file 1 [file Table1.DOCX]

Supporting Information For

**A novel colchicine-myricetin heterozygous molecule: design, synthesis, and effective evaluations on the pathological models of acute lung injury in vitro and in vivo**

**Zhiyue Li^1,2,3,†^, Xueqin Yan^1,2,†^, Jiangchun Wei ^1,2,†^, Liuyang Pu ^1^, Guanbao Zhu^1,4^, Yongkai Cao^1^, Zhanyan Liu^1^*,* Yaqian Liu^1^, Yan Li^1^, Limin Li^1^, Xinping Li^1^, Zhengzhi Wu^1,2,*^**

^1^ Shenzhen Institute of Translational Medicine, The First Affiliated Hospital of Shenzhen University, Shenzhen, China.

^2^ Wu Zhengzhi Academician Workstation, Ningbo College of Health Sciences, Ningbo 315800, China

^3^ Shenzhen Institute of Advanced Technology, Chinese Academy of Sciences, Shenzhen, China

^4^ Guangxi University of Chinese Medicine, Nanning, China.

***Correspondence:**Zhengzhi Wu, Foreign Academician, the National Academy of Engineering Sciences, Ukraine. Email: [szwzz001@163.com](mailto:szwzz001@163.com), , Tel.: 86-755-82530086.

†These authors have contributed equally to this work and share the first authorship.

**Table of Contents**

1. **NMR Spectra…………………………………………………………………….……………......S2**

**1 NMR Spectra**

**Figrue 1. ^1^H NMR Spectra of CMyrH.**

**Figrue 2. ^13^C NMR Spectra of CMyrH.**
